# Monitoring of the lateral spread response combined with brainstem auditory evoked potentials in microvascular decompression for hemifacial spasm

**DOI:** 10.3389/fneur.2025.1516606

**Published:** 2025-06-25

**Authors:** Dejing Cheng, Chengfang Liu, Youjia Qiu, Chengyuan Ji

**Affiliations:** ^1^Department of Rehabilitation, The Forth Affiliated Hospital of Soochow University, Suzhou, China; ^2^Department of Neurosurgery, The First Affiliated Hospital of Soochow University, Suzhou, China

**Keywords:** hemifacial spasm, microvascular decompression, brainstem auditory evoked potentials, lateral spread response, hearing impairment

## Abstract

**Objective:**

Despite high cure rates, symptom persistence and auditory dysfunction occur sometimes after microvascular decompression (MVD) surgery for hemifacial spasm (HFS). This study evaluated whether combined intraoperative monitoring of the lateral spread response (LSR) and brainstem auditory evoked potentials (BAEP) can reduce the incidence of hearing impairment following MVD for HSF.

**Methods:**

A total of 244 HFS patients undergoing MVD were prospectively included and divided into an LSR monitoring group (121 cases) and a combined LRS + BAEP monitoring group (123 cases). Intraoperative recordings of abnormal muscle response (AMR) waves and BAEP were collected and correlated with postoperative HFS and hearing status.

**Results:**

HFS symptoms were similarly improved in the two groups, with no significant differences in the occurrence of AMR or the probability of AMR disappearance postoperatively. For both groups, the sensitivity, specificity, positive predictive value, negative predictive value, and accuracy of AMR waves were also comparable. However, the incidence of postoperative hearing impairment was significantly lower in the LSR + BAEP group compared to the LSR group. Furthermore, receiver operating characteristic (ROC) analysis of BAEP's performance revealed an area under the ROC curve (AUC) of 0.991 (95% CI: 0.955–1.000), indicating a high diagnostic value of BAEP for predicting postoperative hearing decline.

**Conclusion:**

LSR monitoring is a reliable approach for assessing the effectiveness of MVD surgery for the facial nerve. The combination of LSR monitoring with BAEP does not affect diagnostic accuracy. More importantly, BAEP can sensitively reflect patients' hearing changes during surgery due to its high diagnostic value, guiding surgeons to adjust their intraoperative techniques and effectively reducing the incidence of postoperative hearing impairment.

## Introduction

Hemifacial spasm (HFS) is characterized by paroxysmal, involuntary contractions of the muscles on one side of the face innervated by the ipsilateral facial nerve. The most common etiology is compression of the facial nerve's root exit zone at the brainstem by the anterior inferior cerebellar artery. Microvascular decompression (MVD) of the facial nerve is an effective treatment for HFS, with a success rate of over 90% (1). However, the incidence of postoperative complications such as tinnitus, hearing impairment, and even complete hearing loss ranges from 1.9% to 20%, significantly affecting patients' prognosis and quality of life (2, 3).

In MVD surgery for the facial nerve, monitoring the lateral spread response (LSR) has become a routine method to predict postoperative facial spasm symptoms (4). In turn, studies have shown that changes in the latency or amplitude of brainstem auditory evoked potentials (BAEP) waves I and V during MVD for HFS can effectively predict postoperative hearing outcomes (3, 5). However, few studies have reported the combined application of intraoperative LSR and BAEP monitoring for timely adjustment of surgical procedures and prediction of auditory outcomes. Can BAEP monitoring detect early involvement of the vestibular nerve during surgery, where auditory damage may still be reversible? If the surgeon adjusts the procedure promptly, can BAEP signals recover? Could this further reduce the incidence of postoperative hearing loss? Based on these questions, this study compares HFS symptom improvement and hearing status after MVD surgery applying either LSR monitoring alone or in combination with BAEP.

## Materials and methods

### Study design

Based on random digital allocation, patients admitted to the Department of Neurosurgery of the First Affiliated Hospital of Soochow University between January 2022 and December 2023 were divided into two groups: lateral spread response (LSR) monitoring group, and combined LSR + brainstem auditory evoked potentials (BAEP) monitoring group. All enrolled patients signed informed consent forms with the approval of their families, and preoperative evaluations and data collection were conducted. A total of 244 patients with HFS were included in this study over a 2-year period, with 121 cases in the LSR monitoring group and 123 cases in the combined LSR + BAEP monitoring group.

The inclusion criteria were: (1) patients diagnosed with HFS based on preoperative symptoms, physical examination, and imaging data; (2) patients undergoing MVD of the facial nerve for the first time, with intraoperative monitoring using either LSR alone or LSR combined with BAEP; and (3) patients with complete clinical records.

Exclusion criteria: (1) patients who did not undergo surgery; (2) patients with recurrent HFS; (3) patients with preoperative hearing impairment.

### Lateral spread response monitoring

**Figure 1:**
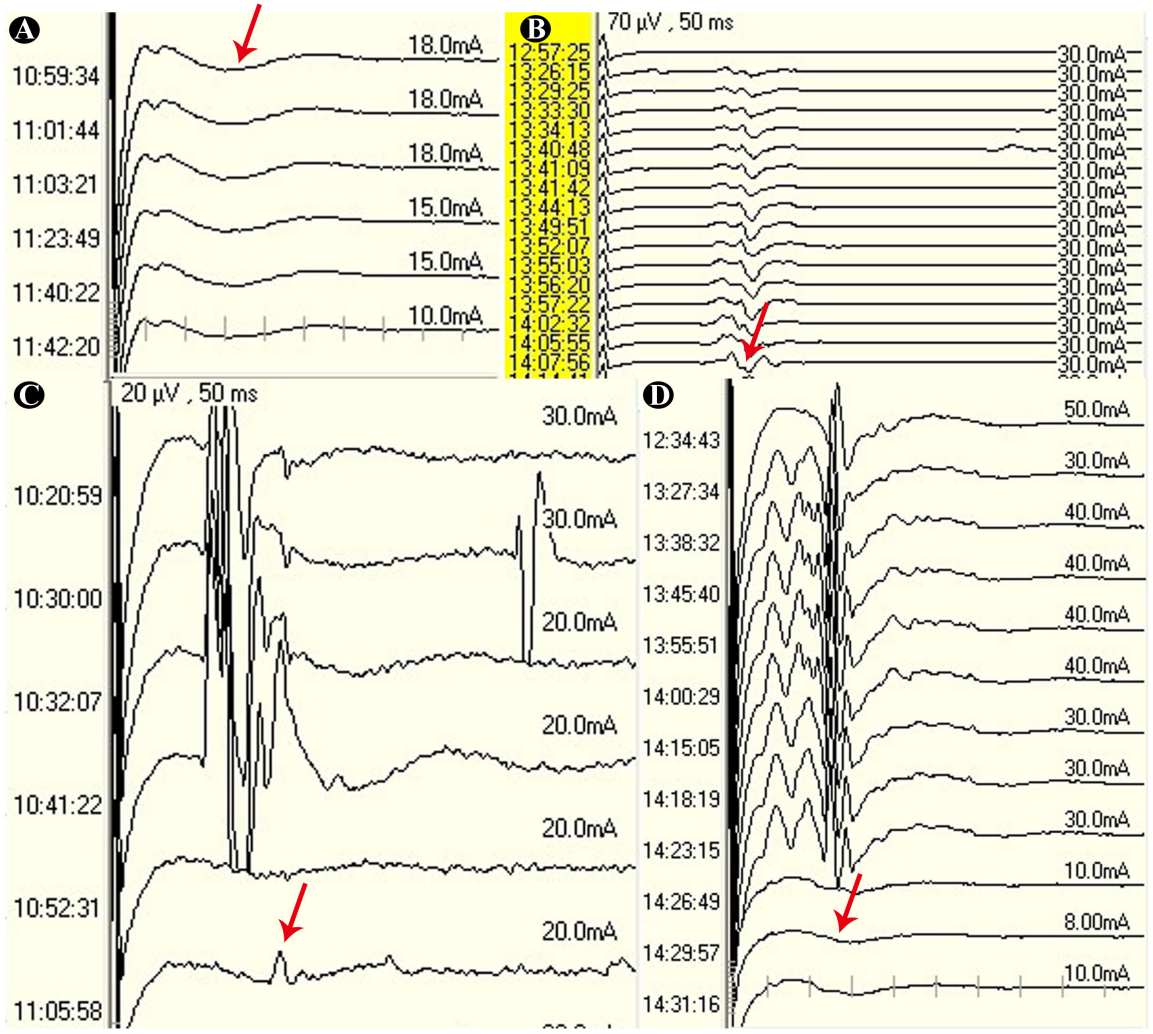
**(A)** The red arrow indicates the absence of the AMR wave; **(B)** the AMR wave is persistently present; **(C)** after the completion of the surgery, the amplitude of the AMR wave decreases by more than 90%; **(D)** after the completion of the surgery, the AMR wave disappears.

Intraoperative neurophysiological monitoring was performed using either a Cadwell (Cadwell Industries, USA) or Nicolet Endeavor CR (Nicolet Biomedical Inc., USA) system. As outlined in Figure 1, stimulation was applied to the zygomatic and mandibular branches of the facial nerve, with abnormal muscle responses (AMR) recorded from the mentalis and orbicularis oculi muscles, respectively. Single-pulse electrical stimulation was used, with a pulse width of 200 μs and a stimulation intensity ranging from 5 to 50 mA. The analysis window was set at 50 ms. The surgeon was promptly informed when the lateral spread response waveform appeared, decreased, or disappeared (6).

We assessed the accuracy of LSR monitoring in predicting HFS outcomes after excluding those patients with no intraoperative AMR waves. Persistent AMR waves (decline < 90%) were defined as persistent AMR, while AMR waves that declined by more than 90% or completely disappeared were defined as disappeared AMR.

### Intraoperative BAEP monitoring

**Figure 2:**
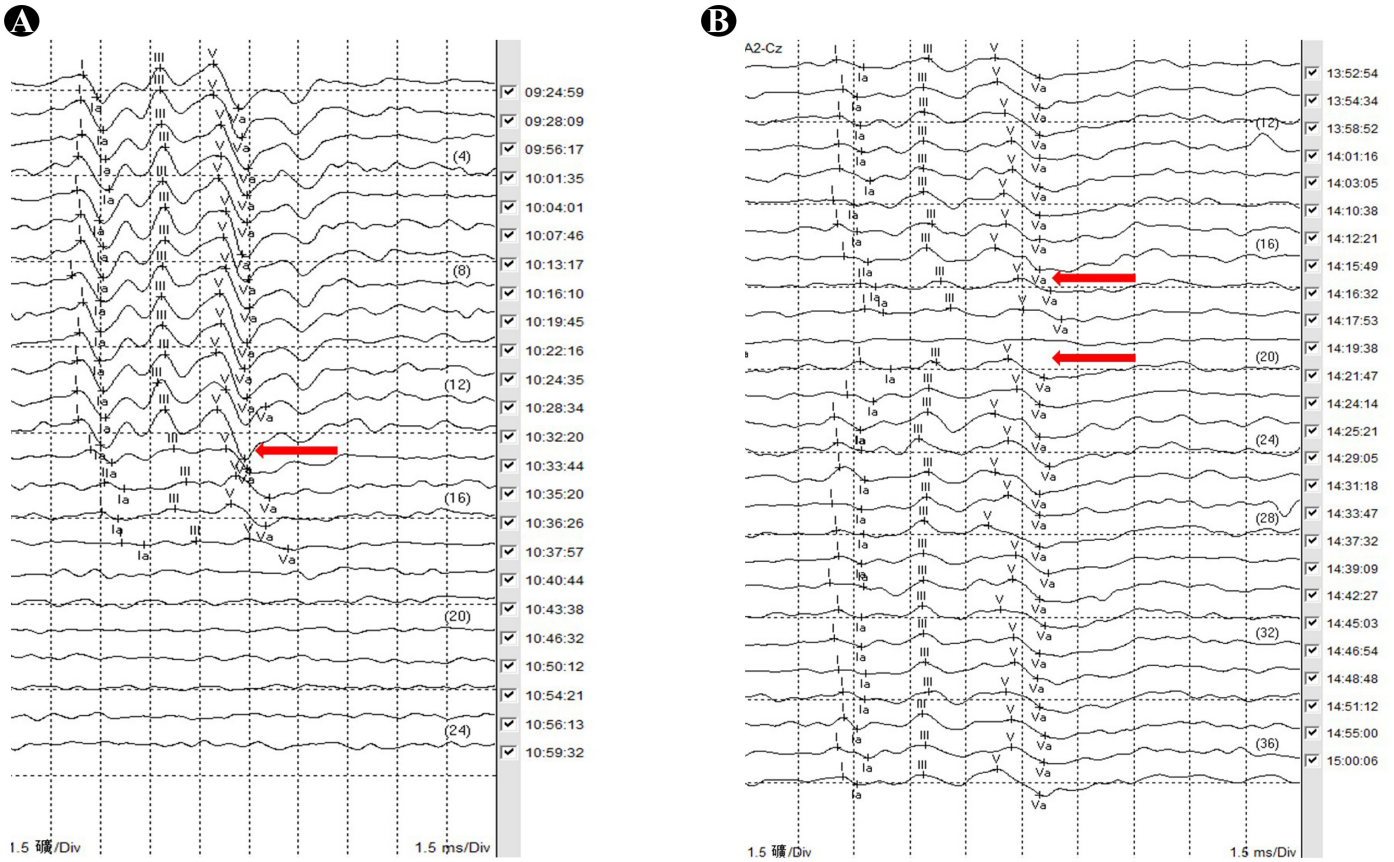
**(A)** At 10:33:44, the amplitudes of waves I and V show a significant decrease, and the latencies are prolonged. At 10:35:20, the amplitudes of waves I and V decreased by more than 50%, with latencies prolonged by more than 0.6 ms. Subsequently, the amplitudes of waves I and V continue to decrease until they disappear; **(B)** at 14:16:32, the amplitudes of waves I and V show a significant decrease, and the latencies are prolonged. At 14:19:38, no distinct waveforms are observed, and by 14:21:47, the waveforms gradually recove.

An earplug was inserted into the external auditory canal on the affected side. The recording electrode (A1/A2) was placed anterior to the tragus, with a reference electrode (CZ) positioned at the vertex. The affected side received short-tone stimuli at a frequency of 11.33 Hz and an intensity of 100 dB. The analysis window was set at 15 ms, with 100 to 1,000 responses averaged (Figure 2). The contralateral side received short-tone stimuli at a frequency of 11.33 Hz, an intensity of 100 dB and with 60 dB white noise masking. The surgeon was alerted when the latency of wave I or V was prolonged by more than 0.6 ms or when the amplitude decreased by more than 50% (7).

We defined no change in BAEP or transient changes in BAEP as unchanged BAEP, while persistent changes in BAEP were classified as changed BAEP. This study aimed to intervene in surgical operations by assessing changes in BAEP during surgery, with the goal of improving hearing outcomes. The transient and permanent changes in BAEP are specific to the surgical procedure. When BAEP changes occur during surgery, electrophysiologists provide timely feedback to the surgical team, allowing them to adjust for neural or vascular manipulation. BAEP amplitudes and/or intervals typically recover within 5 min (possibly extending slightly), which defines a transient change. If BAEP does not recover even after adjustments by the surgical team throughout the procedure, it is considered a permanent change.

### Anesthesia and surgical technique

General anesthesia was administered through a combination of intravenous agents and endotracheal intubation. During anesthesia, we used small oral doses of sevoflurane for inhalation, remifentanil and dexmedetomidine for intravenous administration with sufentanil added intermittently. During surgery, the use of muscle relaxants was minimized, except during the induction phase, to avoid interference with neurophysiological monitoring. Fluid administration was carefully controlled to manage the total volume, and the partial pressure of carbon dioxide (PaCO2) was maintained at ~26 mmHg. β-blockers were used as necessary to facilitate surgical procedures (8).

Whether employing a microscopic or endoscopic approach, MVD of the facial nerve was performed via a retrosigmoid approach. The patient was positioned in the lateral decubitus position with the head securely fixed in a head frame. The head was elevated at an angle of 15°–20°, and the chin was flexed toward the sternum, approximately two fingerbreadths away. The shoulder strap was gently retracted toward the caudal direction to maintain head hyperextension while avoiding excessive traction on the brachial plexus, ensuring that the root of the mastoid process was positioned at the highest point.

**Figure 3:**
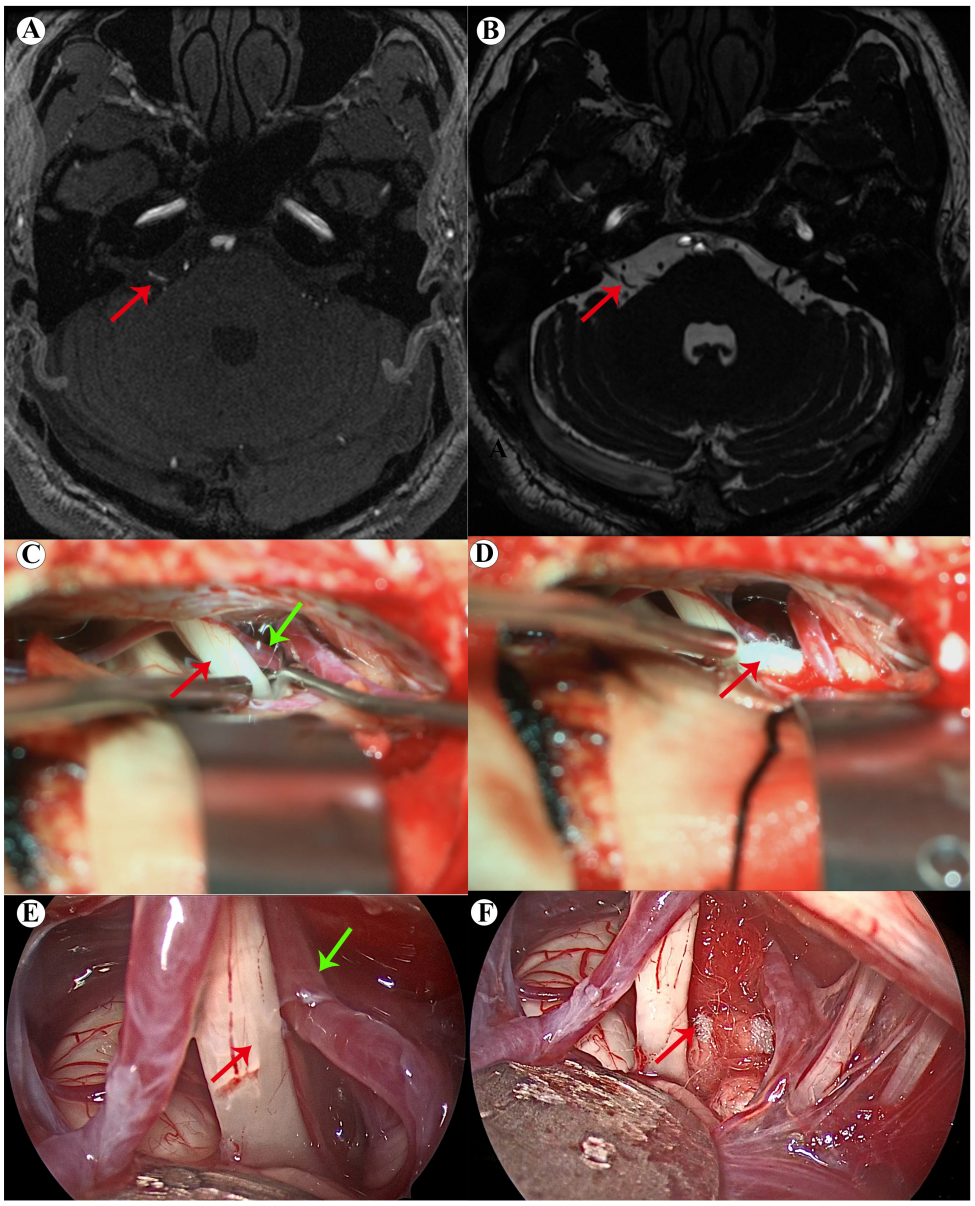
**(A, B)** Preoperative magnetic resonance imaging indicates that the right facial nerve is compressed by the anterior inferior cerebellar artery, with the responsible artery highlighted by the arrow; **(C, D)** microscopic views during surgery; in panel C, the red arrow indicates the facial nerve and the green arrow indicates the responsible artery. In panel D, the red arrow represents the status after the responsible artery has been separated; **(E, F)** endoscopic views during surgery; in panel E, the red arrow indicates the facial nerve and the green arrow indicates the responsible artery. In panel F, the red arrow represents the status after the responsible artery has been separated.

Surgical procedure: the subarachnoid space was accessed to release cerebrospinal fluid (CSF), allowing for a decrease in intracranial pressure. Once the pressure was reduced, a sharp dissection of the arachnoid membrane was performed from the caudal end of the cranial nerves to the rostral end, completely separating the cerebellum from the cranial nerves. The intraoperative exploration focused on the intradural segments of the facial nerve, specifically areas I to IV. If exposure was challenging, endoscopy could be utilized for multi-angle exploration. All vessels in contact with the facial nerve were meticulously dissected and retracted, and appropriate decompression techniques (such as Teflon felt, adhesive agents, or suspension) were employed. Careful release of the arachnoid membrane was crucial to prevent traction on the cranial nerves (Figure 3) (9–11).

**Figure 4:**
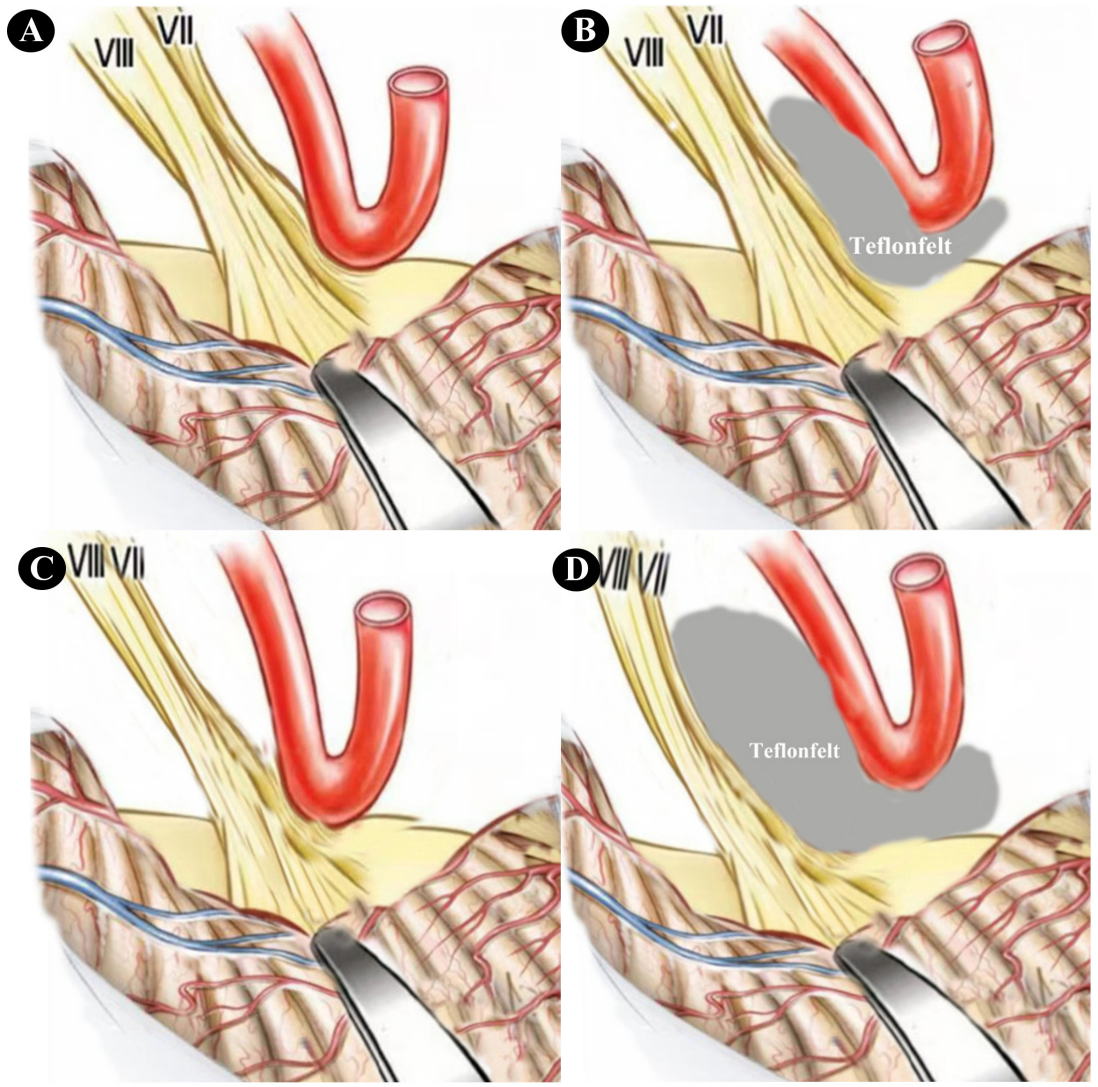
**(A)** Pulling the cerebellar hemisphere posteriorly exposes the VII and VIII cranial nerves and the responsibility artery; **(B)** using Teflonfelt to separate the VII and VIII cranial nerves from the responsibility artery; **(C)** excessive posterior pulling of the cerebellar hemisphere leads to elongation and thinning of the VII and VIII cranial nerves; **(D)** using overly thick or excessive Teflonfelt results in displacement and strain of the VII and VIII cranial nerves.

Surgical technique highlights combined with electrophysiological monitoring: (1) when releasing cerebrospinal fluid, utilize the natural anatomy and gravity of the cerebellum and temporal bone as much as possible while minimizing the use of the brain tractor to avoid excessive traction on the VII and VIII cranial nerves. If changes in BAEP amplitude or latency occur during this process, immediate adjustment of the cranial pressure is necessary. (2) When operating near the blood vessels surrounding the VII and VIII cranial nerves within the brainstem's REZ area, first ensure complete separation of these structures. Perform this step gently to prevent vasospasm or excessive bleeding caused by large operative movements. If BAEP changes during operating blood vessels, the operator must halt the procedure immediately to minimize blood vessel traction or stimulation. Additionally, applying local anesthetics like papaverine on the blood vessel's surface can help relax the vessels and prevent vasospasm if necessary (12). (3) During Telfonfelt reduction, avoid over-filling. If Telfonfelt between blood vessels and nerves is too thick, it may lead to unnecessary nerve traction; thus, monitor BAEP changes during this process to ensure appropriate use of Telfonfelt for effective decompression (Figure 4).

Operation outcome evaluation: We categorized the surgical results as: excellent (E0); good (E1); fair (E2); and poor (E3) (13). E0: Complete disappearance of spasm; E1: Occasional slight spasm; E2: Moderate spasm, apparently persisting; E3: Not cured. Complete recovery and significant improvement were classified as a good outcome, whereas partial relief and no improvement were classified as a poor outcome.

### Statistical analysis

Statistical analyses were conducted using SPSS Statistics version 29.0 and MedCalc version 22.0. Continuous variables were expressed as mean ± standard deviation (*x* ± *s*), while categorical variables were presented as counts and percentages [*n* (%)]. A one-way analysis of variance (ANOVA) was used to compare Karnofsky Performance Scale (KPS) scores among different surgical groups. For comparisons of preoperative and postoperative KPS scores within the same group or between two groups, paired *t*-tests were applied, with results reported as mean differences and 95% confidence intervals (CI). For categorical data analysis, the choice of statistical tests depended on sample size and expected frequencies: Pearson's chi-square test was used when *N* ≥ 40 and expected frequency (*T*) ≥ 5. Continuity-corrected chi-square test was applied when *N* ≥ 40 and 1 ≤ *T* < 5. Fisher's exact test was used for small samples (*N* < 40 or *T* < 1). A *P*-value < 0.05 was considered statistically significant. Receiver operating characteristic (ROC) curve analysis was performed using MedCalc version 22.0 to compare the diagnostic performance of different monitoring methods. The area under the ROC curve (AUC) was calculated to quantify classification performance: AUC = 0.5 suggests no diagnostic ability. AUC < 0.5 indicates performance worse than random guessing. 0.5 ≤ AUC < 0.7 suggests low diagnostic value. 0.7 ≤ AUC < 0.9 indicates moderate diagnostic value. AUC ≥ 0.9 represents high diagnostic value. To compare AUC values between groups, DeLong's test was employed. Sensitivity (true positive rate) represents the proportion of actual positive cases correctly identified, while specificity (true negative rate) indicates the proportion of actual negative cases correctly classified. PPV reflects the proportion of predicted positive cases that are truly positive, whereas NPV represents the proportion of predicted negative cases that are truly negative. Accuracy measures the overall proportion of correctly classified cases among all cases. Statistical comparisons of these metrics were performed using Pearson's chi-square test, continuity-corrected chi-square test, or Fisher's exact test, depending on sample size and expected frequencies. If a particular category within a group had zero positive cases (e.g., no “poor outcome positive” patients in the LSR group), this could lead to extreme values in sensitivity or specificity calculations. In such cases, Fisher's exact test or the continuity-corrected chi-square test was used to mitigate the impact of small or sparse data.

## Results

### General information

A total of 244 patients were included in this study, comprising 93 male and 151 female participants. Among these, 130 patients had left-sided HFS, while 114 had right-sided HFS. The mean age of the patients was 53.7 ± 11.7 years, with a minimum age of 19 years and a maximum age of 83 years. In the LSR monitoring group, 60 patients underwent microscopic surgery, and 61 patients underwent endoscopic surgery. In the LSR + BAEP monitoring group, 64 patients underwent microscopic surgery, and 59 patients underwent endoscopic surgery.

### AMR assessment

In the LSR monitoring group, AMR waves were observed in 117 cases during surgery. Among these, 10 cases exhibited persistent AMR waves, 2 cases showed a decline of more than 90%, and 105 cases had AMR waves that disappeared postoperatively. Four cases did not present any AMR waves. In the LSR + BAEP monitoring group, AMR waves were recorded in 119 cases intraoperatively. Within this group, 9 cases demonstrated persistent AMR waves, 3 cases experienced a decline of more than 90%, and 107 cases had AMR waves that disappeared postoperatively. Four cases in this group did not exhibit any AMR waves. There were no significant statistical differences between the two groups regarding the occurrence and non-occurrence rates of AMR waves or the probability of AMR waves disappearing or remaining postoperatively (Tables 1a,b).

**Table 1a:** The occurrence and non-occurrence rates of AMR wave.

	**LSR alone**		**LSR** + **BAEP**	
**AMR waves during surgery**	**Occurrence rate**	**Non-occurrence rate**	**Occurrence rate**	**Non-occurrence rate**
	117 (96.7%)	4 (3.3%)	119 (96.7%)^**a**^	4 (3.3%)^**b**^

^a, b^The occurrence and non-occurrence rates of AMR wave showed no statistically significant difference between the LSR monitoring group and the combined LSR and BAEP monitoring group (*p* > 0.05, continuity-corrected Chi-square test). LSR: lateral spread response; BAEP: brainstem auditory evoked potentials.

**Table 1b:** The occurrence and non-occurrence rates of the disappearance of AMR wave.

	**LSR alone**			**LSR** + **BAEP**		
**Disappearance of AMR waves after surgery**	**Non-occurrence rate**	**Occurrence rate**		**Non-occurrence rate**	**Occurrence rate**	
	10 (8.5%)	107 (91.5%)		9 (7.6%)^c^	110 (92.4%)^d^	
	**Persisted**	**Decreased by more than 90%**	**Completely disappeared**	**Persisted**	**Decreased by more than 90%**	**Completely disappeared**
	10	2	105	9	3	107

^c, d^Similarly, there was no statistically significant difference in the occurrence and non-occurrence rates of the disappearance of AMR wave between these two groups (*P* > 0.05, Chi-square test). LSR: lateral spread response; BAEP: brainstem auditory evoked potentials.

### Postoperative outcomes

We segregated the intraoperative AMR patterns into four groups (Table 2). In the LSR monitoring group, four cases exhibited no AMR waves, with outcomes of E0 in 2 cases, E1 in 0 cases, E2 in 1 case, and E3 in 1 case. Ten cases demonstrated persistent AMR waves, with results classified as E0 in 4 cases, E1 in 2 cases, E2 in 2 cases, and E3 in 2 cases. Two cases showed a decline in AMR waves of more than 90%, both classified as E0. A total of 105 cases exhibited a complete disappearance of AMR waves, with classifications of E0 in 78 cases and E1 in 27 cases. In turn, in the LSR + BAEP monitoring group, four cases exhibited no AMR waves and outcomes were classified as E0 in 1 case, E1 in 1 case, and E3 in 2 cases. Nine cases demonstrated persistent AMR waves, and outcomes were classified as E0 in 3 cases, E1 in 2 cases, E2 in 2 cases, and E3 in 2 cases. Three cases showed a decline in AMR waves of more than 90%, and were classified as E0 in 2 cases and E2 in one case. A total of 107 cases exhibited a complete disappearance of AMR waves, with classifications of E0 in 79 cases, E1 in 25 cases, E2 in 2 cases, and E3 in 1 case.

**Table 2:** Outcomes of MVD surgery for HFS symptoms.

		**LSR**					**LSR** + **BAEP**				
		**No AMR wave**	**AMR wave persistence**	**AMR wave decreased >90%**	**AMR wave disappearance**	**Total**	**No AMR wave**	**AMR wave persistence**	**AMR wave decreased >90%**	**AMR wave disappearance**	**Total**
Number of patients		4	10	2	105	121	4	9	3	107	123
Outcome	Cured (excellent) E0	2	4	2	78	86	1	3	2	79	85
	Significant relief (good) E1	0	2	0	27	29	1	2	0	25	28
	Partial relief (fair) E2	1	2	0	0	3	0	2	1	2	4
	Ineffective (poor) E3	1	2	0	0	3	2	2	0	1	5
Cure rate		50%	40%	100%	74.3%	71.1%	25%	33.4%	66.7%	73.8%	69.1%
Significant relief rate		0%	20%	0%	25.7%	23.9%	25%	22.2%	0%	23.4%	22.8%
Partial relief rate		25%	20%	0%	0%	2.5%	0%	22.2%	33.3%	1.9%	3.3%
Ineffectiveness rate		25%	20%	0%	0%	2.5%	50%	22.2%	0%	0.9%	4.8%

For both the LSR and LSR + BAEP monitoring groups, no statistical differences in the cure rate were observed between the respective AMR wave disappearance groups and those showing an AMR decline of more than 90%. In contrast, and compared also with the corresponding AMR wave disappearance groups, cure rates were significantly lower in the groups with no AMR waves and those with persistent intraoperative AMR waves (Table 2).

### Diagnostic value of intraoperative monitoring methods for therapeutic effectiveness

Table 3 summarizes the treatment outcomes for the two monitoring methods.

**Table 3:** Comparison of diagnostic value of intraoperative monitoring methods for treatment outcomes.

	**Postoperative outcomes**		**Total**
	**Good outcome (+)**	**Poor outcome (–)**	
**LSR** + **BAEP group**			
Occurrence rate of AMR wave	106	4	110
Non-occurrence rate of AMR wave	5	4	9
Total	111	8	119
**LSR group**			
Occurrence rate of AMR wave	107	0	107
Non-occurrence rate of AMR wave	6	4	10
Total	113	4	117

**Figure 5:**
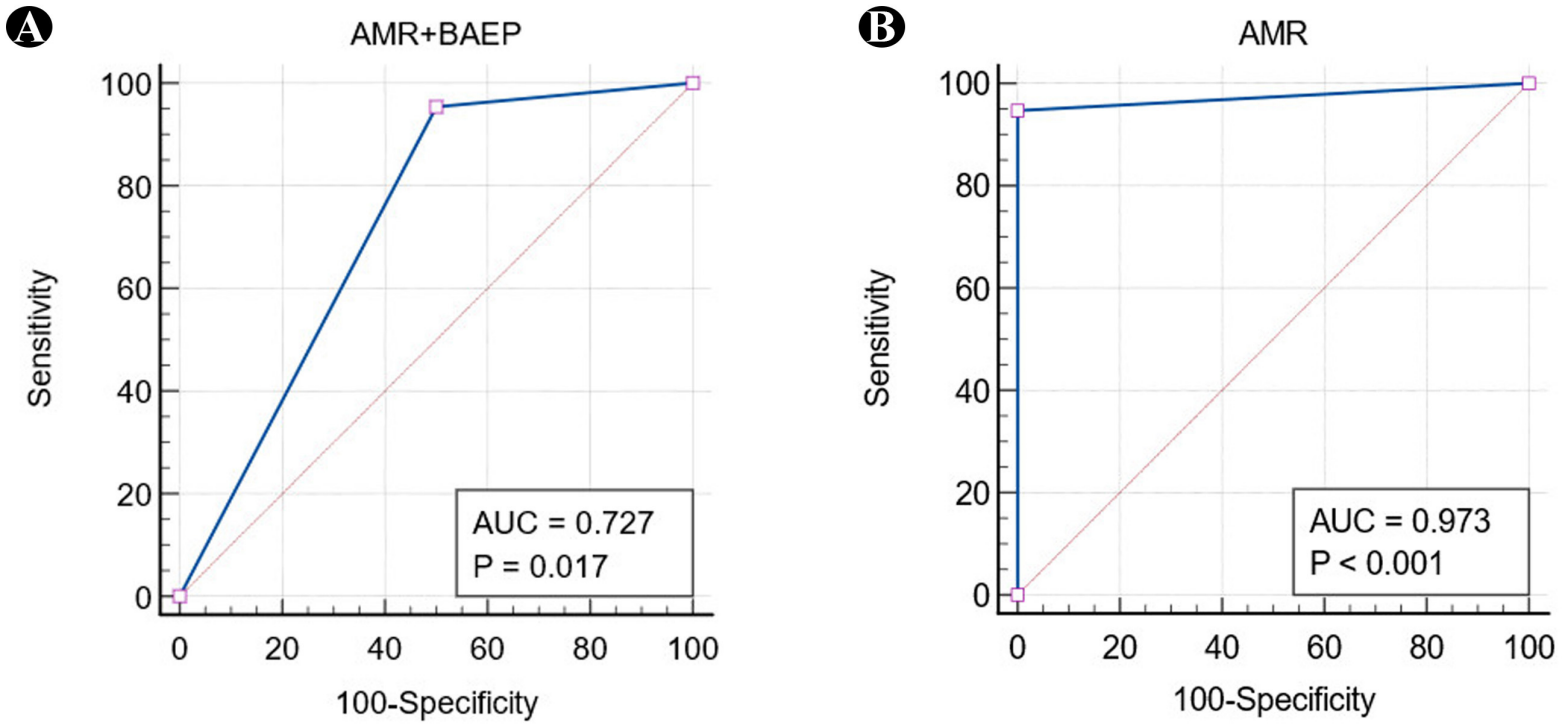
Comparison of ROC curves. **(A)** Indicates an AUC value of 0.727 (95% CI: 0.638–0.805), while **(B)** indicates an AUC value of 0.973 (95% CI: 0.926–0.994).

Based on the postoperative outcomes of both groups, ROC curves were constructed. The AUC for the LSR + BAEP group was 0.727 (95% CI: 0.638–0.805), while the AUC for the LSR group was 0.973 (95% CI: 0.926–0.994). The difference in AUC between the two groups (0.246, 95% CI: 0.0586–0.433) was significant (Figure 5).

The LSR + BAEP group demonstrated a sensitivity of 95.50%, specificity of 50.00%, positive predictive value of 96.36%, negative predictive value of 44.44%, and accuracy of 92.44%. In turn, the LSR group had a sensitivity of 94.69%, specificity of 100.00%, positive predictive value of 100.00%, negative predictive value of 40.00%, and accuracy of 94.87%. Chi-square testing indicated no statistically significant differences among the five diagnostic indicators of the two groups (Table 4).

**Table 4:** Comparison of diagnostic value between two monitoring methods.

	**Sensitivity**	**Specificity**	**Positive predictive value**	**Negative predictive value**	**Accuracy**
LSR + BAEP	95.50%	50.00%	96.36%	44.44%	92.44%
LSR	94.69%	100.00%	100.00%	40.00%	94.87%
χ^2^ value	0.078	–	2.209	–	0.588
*P* value	0.78	0.208	0.137	1.000	0.443

### BAEP-based hearing assessment

In the LSR monitoring group, 16 out of the 121 patients experienced postoperative hearing loss, with an incidence rate of 13.2%, including 2 cases of deafness and 14 cases of hearing impairment. In the LSR + BAEP monitoring group, six patients experienced hearing impairment, resulting in an incidence rate of 4.9%, with no cases of complete hearing loss (Table 5).

**Table 5:** Postoperative hearing assessment.

**Number of patients**		**LSR**	**Incidence rate**	**LSR + BAEP**	**Incidence rate**
No abnormal hearing		105	86.8%	117	95.1%^*^
Abnormal hearing	Surgical side hearing loss	2	13.2%	0	4.9%^**#**^
	Surgical side hearing deterioration	14		6	

^*^Comparing the LSR + BAEP group to the LSR group, a significantly lower incidence of hearing normalities was observed in the latter group (P < 0.05, Chi-square test).

^#^Comparing the LSR + BAEP group to the LSR group, a significantly higher incidence of hearing abnormalities was observed in the latter group (P < 0.05, Chi-square test).

While comparing the LSR + BAEP group to the LSR group alone, the latter exhibited significantly lower normal hearing rates and a higher incidence of hearing abnormalities. This suggests that during MVD surgery to correct HFS, simultaneous BAEP monitoring can reduce the occurrence of postoperative hearing impairment.

### Analysis of the diagnostic value of BAEP

As shown in Table 6, in the BAEP-positive group six patients experienced postoperative hearing loss, while two patients maintained normal hearing. In the unchanged BAEP group, no patients exhibited postoperative hearing loss, with all 115 patients retaining normal hearing.

**Table 6:** Postoperative hearing status of patients in the LSR + BAEP monitoring group.

**BAEP**	**Postoperative hearing status**		**Total**
	**Hearing loss (+)**	**Normal hearing (–)**	
Changed BAEP	6	2	8
Unchanged BAEP	0	115	115
Total	6	117	123

**Figure 6:**
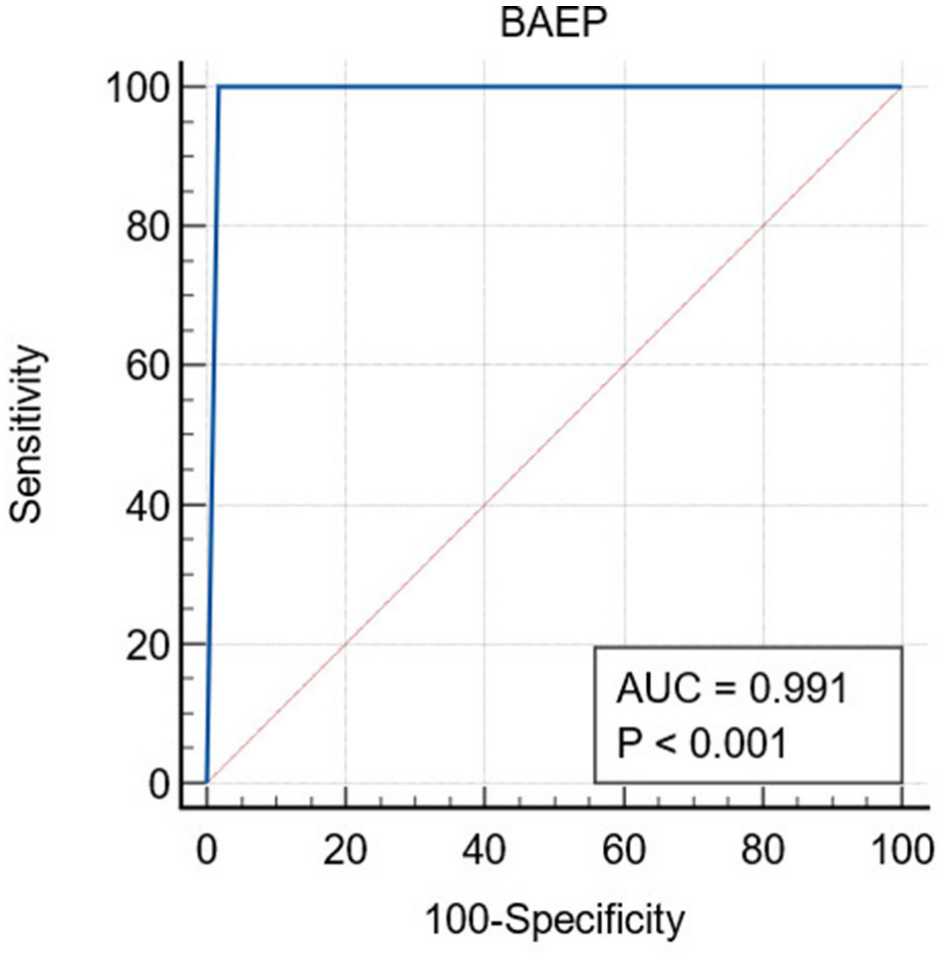
Analysis of the ROC curve for BAEP using MedCalc software indicates an AUC value of 0.991 (95% CI: 0.955–1.000).

From these data, analysis of diagnostic test metrics indicated that sensitivity was 100%; specificity was 98.3%, positive predictive value was 75%, negative predictive value was 100%, and overall accuracy was 95.93%. The AUC ROC for BAEP was 0.991 (95% CI: 0.955–1.000), indicating an excellent diagnostic value for determining postoperative hearing decline (Figure 6).

## Discussion

LSR monitoring provides valuable information for MVD surgery in HFS, as the observation of AMR wave disappearance can be used to predict the effectiveness of the procedure (14). However, few studies have reported the joint application of LSR and BAEP monitoring during surgery to adjust surgical procedures in real time and predict auditory outcomes. Therefore, we divided the prospective cohort into two groups: one group with only LSR monitoring and another group with combined LSR and BAEP monitoring. To establish whether there were differences in the hearing outcomes between the two groups before comparison, we first compared the LSR results from both groups. Our aim was two-fold: to determine if BAEP interfered with LSR and to assess whether there were differences in HFS conditions across the two groups by using LSR as a baseline measurement. Our findings indicated that there were no statistically significant differences in the occurrence and non-occurrence rates of AMR wave between the two monitoring approaches. Likewise, the probability of AMR waves transitioning from disappeared to persistent during surgery was comparable among the two groups. Postoperatively, the rates of complete recovery, significant relief, partial relief, and inefficacy for both monitoring methods also did not demonstrate any significant differences. We thus conclude that BAEP monitoring does not interfere with the assessment of LSR and there was no substantial difference in hemifacial spasm conditions between the two groups of patients.

Currently, LSR is not considered the gold standard for evaluating postoperative recovery or significant symptom improvement; rather, it serves as an adjunctive assessment tool. Our study found that there were no significant differences in sensitivity, specificity, positive predictive value, negative predictive value, and accuracy between the LSR group and the LSR + BAEP group. Regarding the observed AUC differences, we believe these may be influenced by the sparsity of data (i.e., the absence of patients with poor outcomes) in specific categories.

Zero values in ROC AUC calculations can significantly affect the interpretability of model performance. For instance, the absence of “poor outcome positive” patients in the LSR group may lead to an inflated sensitivity (e.g., 100%) and AUC value (0.973), not necessarily reflecting the model's true classification ability. This data sparsity compromises the smoothness and accuracy of ROC curves and may introduce bias when comparing AUCs between groups. Therefore, the observed difference in AUCs might partly result from data imbalance rather than a genuine difference in classification efficacy. To address this limitation, future studies will aim to expand the sample size for more robust analysis. Despite the absence of poor outcome cases in certain LSR subgroups, no significant differences were found between the LSR+BAEP and AMR groups in terms of sensitivity, specificity, PPV, NPV, and accuracy. This can be interpreted from three perspectives: (1) overall data consistency between groups, as most classification categories remained comparable; (2) the use of statistical methods appropriate for small or sparse datasets, such as Fisher's exact test and continuity-corrected chi-square test; and (3) the interrelationship among diagnostic metrics, where improvements in one parameter (e.g., sensitivity) may not lead to significant differences in overall performance due to compensatory changes in others.

Facial nerve MVD surgery primarily utilizes a posterior approach to the sigmoid sinus. The vestibular nerve is located adjacent to the facial nerve, and during the procedure, traction on the cerebellum can lead to stretching or compression of the vestibular nerve due to vascular manipulation. This often results in the vestibular nerve being affected (12), leading to postoperative hearing impairment in patients (2, 5). The changes in latency and/or amplitude of waves I and V of the BAEP can sensitively predict whether a patient will experience hearing impairment following MVD surgery (3).

In the post-operative follow-up of 26 patients with BAEP transient changes, no single case exhibited hearing loss or hearing deterioration. However, this result alone cannot sufficiently demonstrate that our interventions through BAEP modifications and timely warnings to surgeons led to the subsequent recovery of BAEP, thereby preventing postoperative hearing damage. This underscores a critical gap: whether BAEP can effectively track postoperative auditory trends. Only by monitoring BAEP and accurately predicting post-operative hearing status based on its dynamic changes can we provide evidence-based interventions that influence both surgical procedures and ultimate audiometric outcomes.

We further analyzed the diagnostic value of BAEP. The study revealed that combining LSR with BAEP significantly reduced postoperative hearing loss compared to using only LSR. BAEP's high sensitivity (100%) ensures all cases of hearing impairment are detected, and its specificity minimizes false positives. With a strong negative predictive value, a normal BAEP result reliably confirms preserved hearing. These findings underscore BAEP as an effective intraoperative monitoring tool that enhances surgical decision-making by providing early warnings of potential hearing damage, ultimately improving patient outcome.

The tension of blood vessels and cranial nerves VII and VIII near the REZ area is a key factor contributing to postoperative hearing impairment, and BAEP's sensitivity in reflecting hearing changes makes it possible to detect nerve and vessel issues during surgery. When amplitudes or latencies in BAEP measurements change, surgeons should halt operations and assess potential causes—such as vasospasm, blood accumulation from vascular bleeding, and other hemodynamic factors, excessive nerve traction, or Teflonfelt overused (2, 5, 12, 15). Measures like papaverine application or blood washing or reasonable use of tractor to avoid excessive nerve traction and proper use of Teflonfelt should be considered to prevent complications. This study highlights BAEP's diagnostic effectiveness in MVD surgery and the necessity for adjusting surgical procedures based on BAEP changes.

## Conclusions

LSR monitoring is a reliable method for assessing the outcome of MVD surgery for HFS. In combination with BAEP monitoring, the diagnostic accuracy of LSR is unaffected. More importantly, BAEP can sensitively reflect patients' hearing changes during surgery due to its high diagnostic value, guiding surgeons to adjust their intraoperative techniques and effectively reducing the incidence of postoperative hearing impairment.

Of note, this study is a single-center investigation, and due to the limited sample size, the results may be subject to some bias.

## Data Availability

The raw data supporting the conclusions of this article will be made available by the authors, without undue reservation.
